# P-1591. Harnessing Artificial Intelligence for Rapid Interpretation of BCID2 Results: A Pre-Validation Study of a Generative Pre-trained Transformer (GPT)

**DOI:** 10.1093/ofid/ofae631.1758

**Published:** 2025-01-29

**Authors:** Daniel Tassone, Langford Ian, John D Markley, Julia Ye, Matthew M Hitchcock

**Affiliations:** Richmond VA Medical Center, Richmond, Virginia; Virginia Commonwealth University Medical Center, Richmond, Virginia; Central Virginia VA Health Care System, VCU Medical Center, Richmond, Virginia; Central Virginia VA Health Care System, Richmond, Virginia; Richmond VA Medical Center, Richmond, Virginia

## Abstract

**Background:**

Artificial intelligence (AI) has great potential to enhance patient care, but significant questions remain about implementation and safety. Antimicrobial stewardship programs (ASP) could leverage AI to assist with prospective audit and feedback (PAF) interventions such as interpretation of blood culture results and rapid optimization of antimicrobials. At our institution, PAF interventions are performed by the ASP as soon as possible when BioFire® Blood Culture Identification 2 Panel (BCID2) results are available. However, these interventions are time consuming and often delayed due to limited resources. To address this gap, we developed a Generative Pre-trained Transformer (GPT) tool to assist non-infectious disease (ID) providers with interpretation of BCID2 results. Herein, we aim to describe the process of training the GPT and report pilot study results of the tool’s accuracy compared with ASP recommendations.

**Figure:**
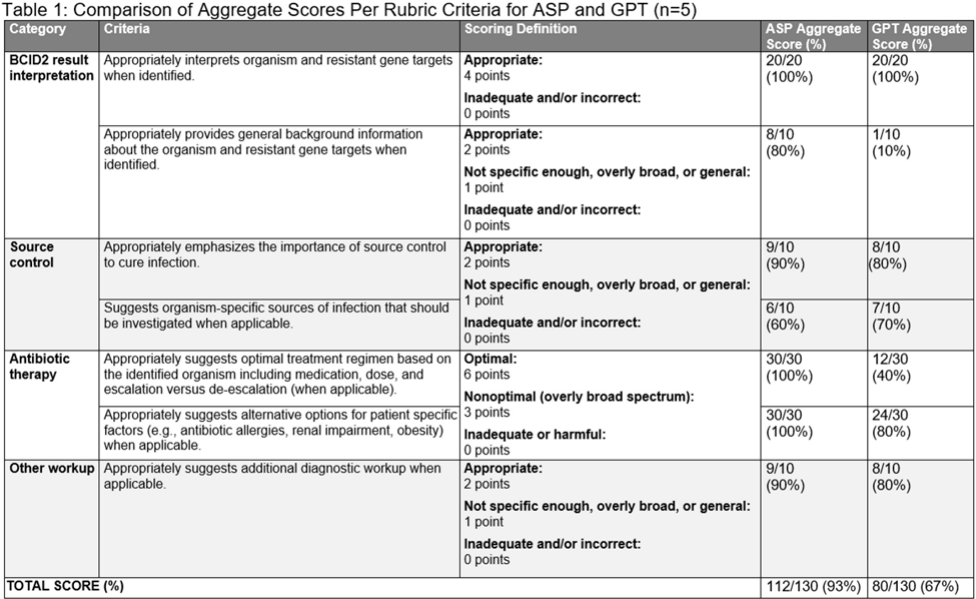

**Methods:**

Using OpenAI’s GPT-4.0, our GPT was trained to synthesize patient-specific treatment advice by analyzing BCID2 results and anonymized patient data. Training focused on applying local as well as national evidence-based recommendations for the management of bacteremia, and providing feedback to the GPT when errors were recognized. A scoring rubric was designed to compare GPT treatment recommendations to gold standard ASP recommendations for the management of bacteremia (Figure 1). An independent infectious disease expert reviewer applied the rubric to determine the accuracy of the GPTs recommendations.

**Figure:**
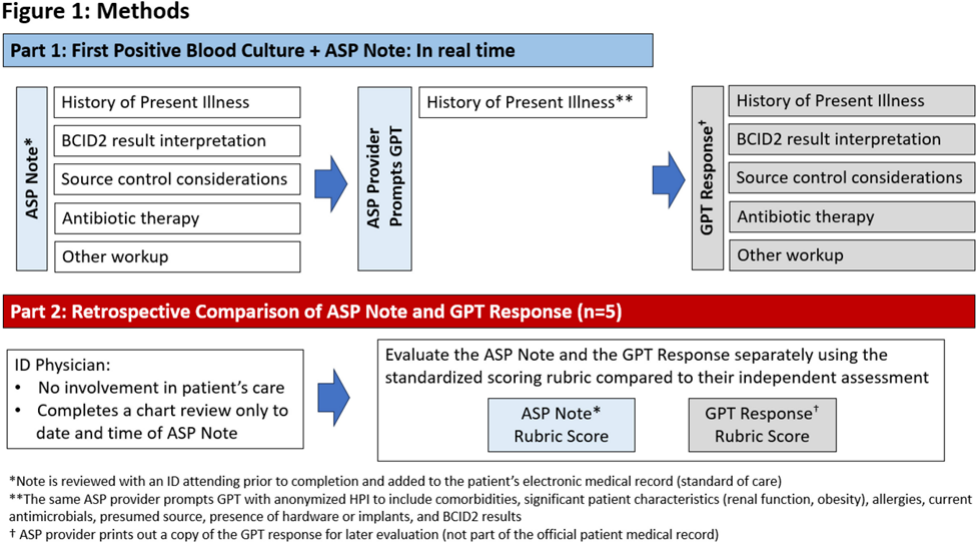

**Results:**

Five cases of bacteremia were retrospectively reviewed. The GPT recommendations were 67% concordant with best practices, compared to 93% for the ASP (Table 1).

**Conclusion:**

In this pilot study, a GPT was trained to interpret and manage bacteremia and achieved 67% concordance with best practices. Although inferior to ASP recommendations, GPT is a promising AI tool that warrants further investigation to aid in ASP interventions. Our GPT has been retrained based on our findings, and a larger prospective validation study is underway.

**Disclosures:**

**Daniel Tassone, PharmD, BCIDP**, Merck: Honoraria

